# Uncovering the Rare Variants of *DLC1* Isoform 1 and Their Functional Effects in a Chinese Sporadic Congenital Heart Disease Cohort

**DOI:** 10.1371/journal.pone.0090215

**Published:** 2014-02-28

**Authors:** Bin Lin, Yufeng Wang, Zhen Wang, Huilian Tan, Xianghua Kong, Yang Shu, Yuchao Zhang, Yun Huang, Yufei Zhu, Heng Xu, Zhiqiang Wang, Ping Wang, Guang Ning, Xiangyin Kong, Guohong Hu, Landian Hu

**Affiliations:** 1 The Key Laboratory of Stem Cell Biology, Institute of Health Sciences, Shanghai Jiao Tong University School of Medicine (SJTUSM) and Shanghai Institutes for Biological Sciences (SIBS), Chinese Academy of Sciences (CAS), Shanghai, People's Republic of China; 2 Diagnosis and Treatment Center of Congenital Heart Disease, the First Hospital of Hebei Medical University, Shijiazhuang, Hebei, People's Republic of China; 3 Clinical Laboratory, Affiliated Hospital of Binzhou Medical College, Binzhou, Shandong, People's Republic of China; 4 State Key Laboratory of Medical Genomics, Ruijin Hospital Affiliated to Shanghai Jiao Tong University School of Medicine, Shanghai, People's Republic of China; Kunming Institute of Zoology, Chinese Academy of Sciences, China

## Abstract

Congenital heart disease (CHD) is the most common birth defect affecting the structure and function of fetal hearts. Despite decades of extensive studies, the genetic mechanism of sporadic CHD remains obscure. Deleted in liver cancer 1 (*DLC1*) gene, encoding a GTPase-activating protein, is highly expressed in heart and essential for heart development according to the knowledge of *Dlc1*-deficient mice. To determine whether *DLC1* is a susceptibility gene for sporadic CHD, we sequenced the coding region of *DLC1* isoform 1 in 151 sporadic CHD patients and identified 13 non-synonymous rare variants (including 6 private variants) in the case cohort. Importantly, these rare variants (8/13) were enriched in the N-terminal region of the DLC1 isoform 1 protein. Seven of eight amino acids at the N-terminal variant positions were conserved among the primates. Among the 9 rare variants that were predicted as “damaging”, five were located at the N-terminal region. Ensuing *in vitro* functional assays showed that three private variants (Met360Lys, Glu418Lys and Asp554Val) impaired the ability of DLC1 to inhibit cell migration or altered the subcellular location of the protein compared to wild-type DLC1 isoform 1. These data suggest that *DLC1* might act as a CHD-associated gene in addition to its role as a tumor suppressor in cancer.

## Introduction

Congenital heart disease (CHD) presents a variety of structural malformations of the heart or great vessels at birth, constituting a major cause of birth defect-related deaths [1]. Although decades of research have revealed that both environmental and genetic factors contribute to the etiology of CHD, increasing evidence supports an important role of a genetic predisposition to the disease [1]–[4]. Indeed, many disease-causing genes, which follow Mendelian patterns of inheritance (e.g., *TBX5*, *JAG1*, *NKX2-5*, *GATA4*, *NOTCH1*), have been identified by pedigree analysis [5]–[10]; however, the genetic mechanism of most sporadic CHD cases remains elusive [11].

In our previous mutational screen in a Chinese sporadic CHD cohort, a low-coverage (100×) exome sequencing of 18 pooled samples identified a splice-site mutation (chr8:13072284, C>G, reference assembly: hg19) of the deleted in liver cancer 1 (*DLC1*) gene in a patient who has atrial septal defect (ASD). This variant is not recorded in The 1000 Genomes Project database and the dbSNP 137 database; after validation assays, it is absent in 800 control samples, suggesting that this splicing site mutation is unique in the CHD cohort (unpublished data).

*DLC1*, which encodes a GTPase-activating protein, is considered to be a tumor suppressor gene in several types of tumors (e.g., primary hepatocellular carcinoma, breast cancer, prostate cancer, non-small cell lung carcinoma and meningioma tumors) [12]–[18]. The migration and proliferation of some tumor cells are reported to be inhibited by DLC1 [19]–[22]. DLC1 can interact with tensin family proteins [23], [24] and is localized to focal adhesions [25], which together indicate that DLC1 is essential for the cytoskeletal organization and morphology of cells. Interestingly, *Dlc1* ^−/−^ mice are embryonic lethal, and histologically, the heart is incompletely developed with a distorted architecture of the chambers [26]. Another study reported that *Dlc1* homozygous gene-trapped mice demonstrated abnormalities in the embryonic heart and blood vasculature of the yolk sac [27]. These results, which were derived from observations of knockout mice, unequivocally prove that *DLC1* is of paramount importance to the developmental events occurring in the embryonic heart.

The human *DLC1* gene encodes four transcript variants: isoforms 1–4 encode protein products of 1528 aa, 1091 aa, 463 aa and 1017 aa, respectively. Although there have been numerous investigations focused on characterizing the multi-faceted function of *DLC1* isoform 2, the properties of the other isoforms remain unclear. In particular, *DLC1* isoform 1, the longest isoform of the *DLC1* gene (NCBI Reference Sequence: NM_182643.2), is abundantly expressed in human heart tissues [28].

The evidence described above logically leads to the hypothesis that, in addition to its role as a tumor suppressor in cancer, DLC1 might play another role in the pathogenesis of CHD. Therefore, to verify the rare variant frequency of *DLC1* isoform 1 in a CHD cohort, we sequenced the coding regions and intron boundaries of *DLC1* isoform 1 in 151 CHD patients (not including the initial screening CHD cohort of our previous work). Functional experiments were then performed to determine the consequences of the identified mutations.

## Materials and Methods

### Ethics statement

The written informed consent for the genetic analysis was obtained from all the subjects who participated in this study, and the research was approved by the ethics committee at Institute of Health Sciences, Shanghai Institutes for Biological Sciences, Chinese Academy of Sciences.

### Sample preparation

A total of 151 patients with congenital heart disease were enrolled in the study at the First Hospital of Hebei Medical University. All the subjects were examined by experienced cardiologists, and the cardiac phenotypes were determined using standard transthoracic echocardiography and other tests according to the ICD-10 diagnostic criteria (Table S1 in File S1). The patients' basic medical situation and family history were recorded. The karyotypes of all patients were examined; with the exception of three individuals with trisomy 21, all others were normal. Most of the patients did not have extra-cardiac manifestations except the three individuals with Down syndrome. Most of the patients had undergone cardiac catheterization or surgery. After recruitment in Hebei and Shanghai of normal individuals without CHD, control blood samples (*n* = 500) were collected. Genomic DNA was extracted from peripheral blood using QIAamp DNA Blood Mini Kits.

### Mutational analysis

The exons and portions of 5′UTR and 3′UTR regions of *DLC1* isoform 1 were amplified using the primers shown in Table S2 in File S1. The PCR products were then purified using ExoSAP-IT reagent (USB) and sequenced with an ABI 3730 Genetic Analyzer. The results were analyzed using the ABI software suite and the identified variants were re-sequenced and validated.

### Mutation simulation

The method of O'Roak *et al*. [29] was used to calculate the mutation weight of each base of the *DLC1* isoform 1 coding sequence. Because the simulation only focused on the *DLC1* gene, the locus-specific substitution rate was not considered. Thus the mutation weight for each base and each substitution can be calculated as follows:
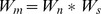
where W_n_ is the weight measuring the nucleotide-specific substitution rates and has two values according to the base composition [30]:
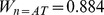


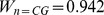



For the weight W_s_, which represents the relative transition or transversion substitution rates [31]:
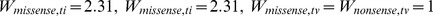



We mutated each base to the other three bases and predicted the class of mutation (i.e., synonymous, missense or nonsense) that would be introduced. For the sake of convenience, only the missense and nonsense classes were considered. We then obtained the mutation weight of each base for missense and nonsense classes using:
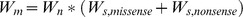



To address whether the cluster of mutations we observed was identical to that expected by chance, after the common SNP sites were eliminated from the coding sequence, 13 non-synonymous rare mutations were randomly introduced into the gene based on the mutation weights in one simulation. We then recorded how often the number of mutations residing within the identical range of our cluster was larger than or equal to 8. The range of the cluster was defined as 639 bp (the length from substitution Ala220Val to Thr433Asn in the coding sequence). The significance was estimated as 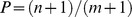, where *n* is the number of instances where the randomized number was greater than the observed number and *m* was the number of randomizations (we employed 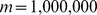). Thus, we could estimate the probability of the identical cluster occurring by chance.

### Plasmids construction

The wild-type *DLC1* isoform 1 expression plasmid was purchased from OpenBiosystems. Seven missense mutants of *DLC1* isoform 1 (threonine substitution of alanine-350, lysine substitution of methionine-360, methionine substitution of leucine-413, lysine substitution of glutamic acid-418, valine substitution of aspartic acid-554, valine substitution of leucine-952 and leucine substitution of valine-1371) were generated by site-directed mutagenesis. The wild type *DLC1* isoform 1 and these mutants were cloned into the pEGFP(N1) plasmid, and the DLC1-GFP fusion constructs were transferred into the retroviral plasmid pBabe-puro.

### Cell culture

The human umbilical vein endothelial cell line (HUVEC, acquired from Lonza) was maintained in basal medium 199 (Invitrogen) with 20% fetal bovine serum (FBS), heparin (25 µg/mL, Sigma) and endothelial cell growth supplement (ECGS) (50 µg/mL, Sigma). The human bone marrow endothelial cell line (HBMEC-60) [32] was maintained in basal medium 200 (Invitrogen) with 20% FBS and a low-serum growth supplement (Invitrogen). The amphotropic Phenix packaging cell line, H29 was maintained in Dulbecco's modified Eagle's medium (DMEM, Invitrogen) with 10% FBS (HyClone), 100 units/mL penicillin, 100 µg/mL streptomycin (Invitrogen) and 1 µg/mL tetracycline (Sigma) in 5% CO_2_ at 37°C.

### Transwell migration assay

To test the effects of the DLC1 wild-type and mutant proteins on cell migration, pBabe-puro overexpression plasmids were transfected into the amphotropic Phenix packaging cell line, and the viruses were collected as previously described [32]. When the cells (HUVEC or HBMEC-60) grew to 30∼40% confluency, the culture medium was replaced with a 1∶1 mixture of fresh medium and the above virus-containing medium in the presence of 5 µg/mL polybrene for infection and this operation was repeated every 24 h until the infection rate of the target cells reached ∼80%, as judged by GFP-positive cells. After infection, 10^5^ infected endothelial cells were resuspended in fresh media containing 0.5% serum, and the cells were seeded in inserts (Costar) containing 8 µm pores. These inserts were placed in Transwell cartridges that contained 300 µL of medium with 10% FBS in the bottom wells. At 24 h after seeding, the medium was aspirated, and 350 µL of trypsin was added into the wells to trypsinize the cells that had passed through the pores. After serum neutralization of the trypsin, the trypsinized cells were centrifuged for 4 min at 1000 rpm, resuspended in 100 µL phosphate-buffered saline (PBS) and counted using a hemocytometer.

### Proliferation assay

When the virus infection rate reached ∼80%, 5×10^4^ infected cells were seeded. After 2 days, the resulting cells were trypsinized and counted using a hemocytometer. Then, 5×10^4^ of these cells were reseeded for another round of counting. The process was repeated for at least three cycles.

### Active rho assay

Cells at 80% confluence were gently rinsed once with ice-cold Tris-buffered saline (TBS) and lysed. The lysate was centrifuged at 16,000×g at 4°C for 15 min, and the supernatant was subjected to active Rho purification and detection with the Active Rho Kit (Pierce, Cat No. 16116) according to the manufacturer's protocol.

### Stress fiber staining and DLC1 subcellular localization

When the cells reached 40% confluence, they were transfected with pEGFP(N1) plasmids harboring *DLC1* wild-type or mutant cDNA. After 24 h, the cells were fixed with 10% formalin for 15 min, permeated with 0.1% Triton X-100 for 10 min and stained with 5 units/mL rhodamine phalloidin (Invitrogen) for 20 min. The stained cells were imaged with using a laser confocal microscope. A total of 100 randomly selected transfected cells in each sample were assessed for subcellular localization of the DLC1-GFP fusion protein. The selected cells were also assessed for the percentage of cells with visible stress fibers as previously described [33].

### Angiogenesis (tube-formation) assay

A total of 5×10^4^ cells infected with *DLC1*-expressing viruses were suspended in 300 µL of DMEM supplemented with 10% FBS and 10 ng/mL FGF (Invitrogen). The cell suspension was seeded on 300 µL of pregelled Matrigel (10.8 mg/mL, Becton, Dickinson and Company). After 24 h, 10 microscopic fields were randomly selected for each well. Angiogenesis in each well was determined by counting the branch points of the formed tubes, as previously described [34].

### Apoptosis assay

Cell apoptosis analysis was performed using an Apoptosis Assay Kit (Keygen Biotech) according to the manufacturer's instructions. Briefly, 1×10^6^ cells infected with virus expressing wild-type or mutant *DLC1* were trypsinized and resuspended in 500 µL of 1× binding buffer. Then, fluorochrome-conjugated Annexin V was added to the cell suspension and was incubated for 10 min at room temperature, followed by incubation with 5 µL of 7-AAD viability staining solution for 10 min at room temperature. The cells were then subjected to flow cytometry using a FACSAria (BD Biosciences).

## Results

### Identification of rare variants in the *DLC1* gene of CHD patients

**Figure 1:**
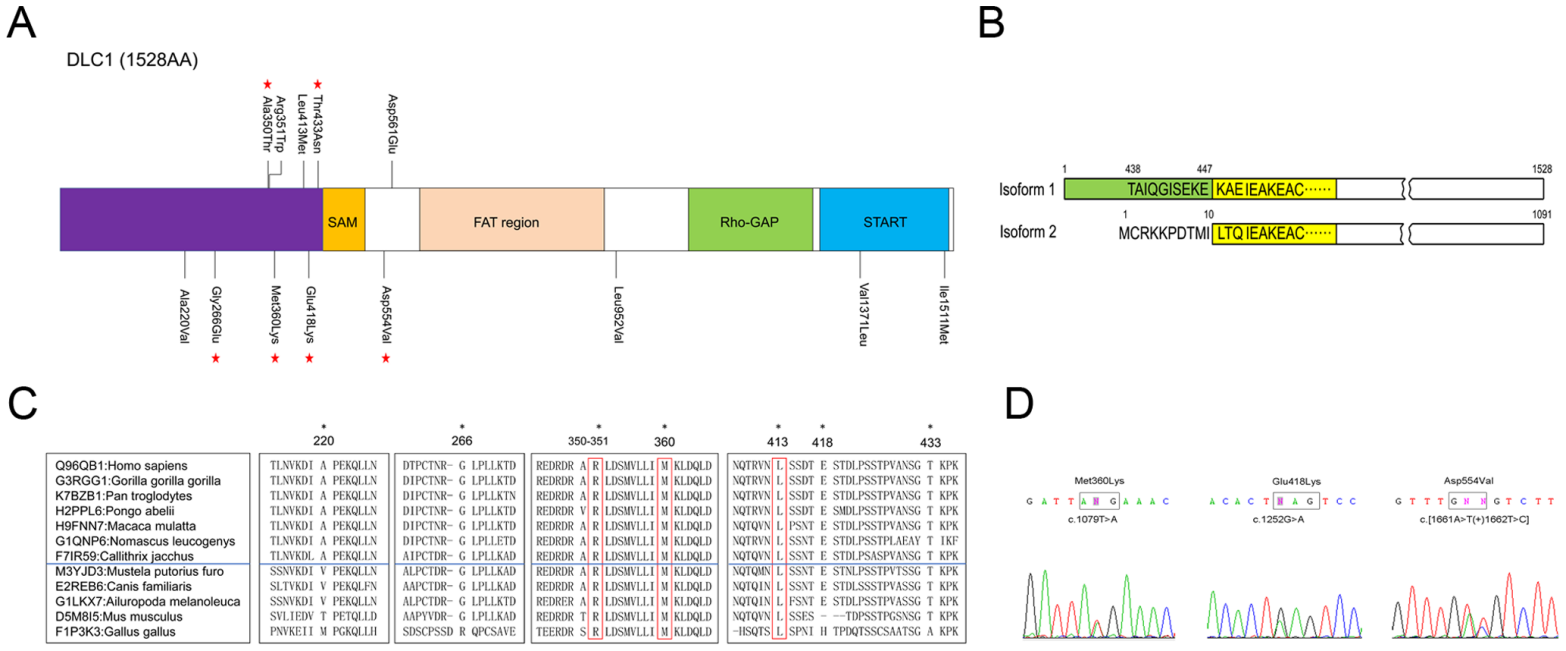
Rare variants identified in *DLC1* isoform 1. (A) The locations of the rare variants are indicated by black lines on the DLC1 isoform 1 protein. FAT (focal adhesion targeting) region, SAM (sterile alpha motif), Rho-Gap (Rho-GTPase-activating protein) and START (steroidogenic acute regulatory protein related lipid transfer) domains are indicated by different colors. Stars denote the private variants identified in the CHD cohort. (B) DLC1 isoform 1 possesses an extended N-terminal region compared to isoform 2. The first 437 residues of isoform 1 are missing in isoform 2, and the sequence ‘TAIQGISEKEKAE’ is replaced by ‘MCRKKPDTMILTQ’ in isoform 2. The yellow box indicates the SAM domain in DLC1, and the green box shows the N-terminal region. (C) The conservation of residues in the N-terminal region was analyzed in different species. The primates and non-primates are separated by the blue lines in the boxes. Asterisks indicate the residues that are conserved among the primates. The residues that are conserved in the primates and non-primates locate in the red boxes. The UniProt accession ID is followed by a colon and the corresponding species name. (D) The private variants that altered the regulation of cell migration function of DLC1 are shown.

*DLC1* isoform 1 contains 18 exons and spans 431,558 base pairs (bp). Each exon of *DLC1* isoform 1 was amplified from the genomic DNA of 151 CHD patients and the PCR products were then sequenced by Sanger sequencing. After eliminating the common single-nucleotide polymorphisms (SNPs) (SNPs with minor allele frequency 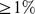) found in the dbSNP database, 13 rare non-synonymous variants were identified. One of these variants was found in 2 patients and each of the rest 12 variant was found in 1 patient. We then assessed the frequency of these rare variants in the control cohort by sequencing the corresponding sites in 500 normal samples using Sanger sequencing method. These data were combined with an additional exome sequencing dataset of 400 individuals (average depth 60×) (G.N., unpublished data) to widen the control cohort to 900 individuals. Consequently, only 3 rare variants identified in the CHD cohort were also found in the controls. In addition, 6 of the 13 variants were SNPs with very low frequency recorded in dbSNP build 137 (Table 1). Altogether, we identified 6 private variants that were absent in 900 controls and the dbSNP database (Table 1, Fig. 1A). The clinical information of 14 patients who carried these rare variants of *DLC1* were reviewed, and ten of the fourteen patients had septal defects. We also reviewed the health status information of the parents of these patients, and all of them had no cardiac defects. However, it's a great pity that we could not obtained the blood samples of these parents because they came to the hospital years ago and we lost touch with these families.

**Table 1:** The rare variants identified in *DLC1* isoform 1.

Variant type	Patient ID	Gender	Age of diagnosis	Diagnosis	Exon	Nucleotide alteration^a^	Amino acid alteration	SIFT score	SIFT prediction ( 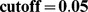 )	Number of mutations in patients	Number of mutations in controls	In dbSNP^b^	ALT allele frequency in dbSNP^c^
Private variants	67	M	5	VSD&PFO	2	c.797G>A	p.Gly266Glu	0.406	Tolerated	1/151	0/900	Na	Na
	153	F	8	VSD	3	c.1048G>A*	p.Ala350Thr	0.368	Tolerated	1/151	0/900	Na	Na
	168	F	5	ASD	3	c.1079T>A*	p.Met360Lys	0.001	Damaging	1/151	0/900	Na	Na
	169	F	22	PS	4	c.1252G>A*	p.Glu418Lys	0.027	Damaging	1/151	0/900	Na	Na
	89	F	2	PDA	4	c.1298C>A	p.Thr433Asn	0.02	Damaging	1/151	0/900	Na	Na
	131	F	8	PDA	9	c.[1661A>T(+)1662T>C]*	p.Asp554Val	0.014	Damaging	1/151	0/900	Na	Na
	190	F	7	VSD	9	c.[1661A>T(+)1662T>C]*	p.Asp554Val	0.014	Damaging	1/151	0/900	Na	Na
Other rare variants	49	F	9	TOF	2	c.659C>T	p.Ala220Val	1	Tolerated	1/151	1/900	Na	Na
	61	F	6	TOF	3	c.1051C>T	p.Arg351Trp	0	Damaging	1/151	2/900	rs144283917	2.324/5869
	42	F	17	VSD	4	c.1237T>A*	p.Leu413Met	0.005	Damaging	1/151	0/900	rs143447199	1/4545
	55	F	26	PDA	9	c.1683C>A	p.Asp561Glu	0.171	Tolerated	1/151	2/900	rs201661577	5/2174
	124	F	4	VSD	9	c.2854C>G*	p.Leu952Val	0.003	Damaging	1/151	0/900	rs184157214	1/2000
	28	M	1	VSD	16	c.4111G>C*	p.Val1371Leu	0.016	Damaging	1/151	0/900	rs142865083	1/2000
	8	M	12	VSD	18	c.4533C>G	p.Ile1511Met	0.001	Damaging	1/151	0/900	rs78322853	Na

Note. Na, no available data; M, male; F, female; VSD, ventricular septal defect; PFO, patent foramen ovale; ASD, atrial septal defect; PS, pulmonary stenosis; PDA, patent ductus arteriosus; TOF, tetralogy of Fallot. a, Nucleotide numbering is according to the RefSeq database NM_182643.2. b, The version of dbSNP used in the table is dbSNP build 137. c, The alternative allele frequency form the dbSNP database is calculated by the alternative allele count/two times the number of individuals assayed. *The mutant vectors were constructed according to these variants.

### *DLC1* rare variants cluster in the N-terminus of the protein

Compared to DLC1 isoform 2, which is the most studied isoform, the coding product of isoform 1 has an N-terminal end of 447 amino acids prior to the SAM domain (including an extended region of 437 amino acids and 10 amino acids which are different from the corresponding parts of DLC1 isoform 2) (Fig. 1B). Although several domains have been identified in the DLC1 protein, the function of the N-terminus is still undefined. Interestingly, 8 (61.5%) of the amino acid-altering variants identified in sporadic CHD were located in this region (Fig. 1A). To evaluate the rare variant frequency of this region in other populations, the rare variant information of *DLC1* in the 1000 Genomes Project [35] and the Exome Sequencing Project [31] were collected and analyzed (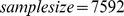). As described before, we defined amino acids 1-447 as the N-terminal region and found that 60 (29.6%) of the 203 rare protein-altering variants were localized in this region (Table S3 in File S1). Consequently, Fisher's exact test (two-tail) showed that, compared to variants found in the 1000 Genomes Project and the Exome Sequencing Project mentioned above, the rare variants identified in our CHD cohort significantly clustered at the N-terminus (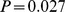), revealing that this might be a disease-associated mutation hot spot. We then used the methods from O'Roak *et al*. [29] to measure the mutation weight of each base of the *DLC1* isoform 1 coding sequence. Subsequently 13 missense or nonsense mutations were randomly introduced into the gene in a simulation according to the mutation weights. After one million simulations, we found that the probability of mutation enrichment similar to the observed cases (at least 8 mutations in a range of 639 bp) was very low (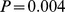), which illustrated that the existence of this mutation cluster in the case cohort was not a spontaneous phenomenon.

### Most rare variants are predicted to be deleterious

We then BLAST-searched the N-terminal sequence in the UniProt database and aligned the homologous sequences [36]. The alignment showed that, seven of eight amino acids at the N-terminal variant positions were conserved among the primates, and it's worth noting that Arg351, Met360 and Leu413 were conserved in the primates and non-primates (Fig. 1C). The SIFT scores were also calculated to predict the effects of the rare variants on protein function [37] (Table 1, Table S3 in File S1). Among the 9 rare variants that were predicted as “damaging” in the case cohort (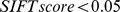), 5 were located at the N-terminal region. As for other five rare variants beyond the N-terminal end, there were three amino acid substitutions in the region between the sterile alpha motif (SAM) and Rho-GTPase-activating protein (GAP) domains, but none in the focal adhesion targeting region [38], [39]. The other two amino acid substitutions (Val1371Leu and Ile1511Met) were located in the steroidogenic acute regulatory protein related lipid transfer (START) domain. All of these substitutions were predicted to be deleterious except the c.1683C>A transition (Table 1). We also evaluated the effects of these 13 rare variants found in the case cohort by multiple prediction methods (PolyPhen-2, LRT, Mutation Taster, etc.), and the prediction results from PolyPhen-2 were similar to the SIFT results (Table S4 in File S1).

### Three mutations affect the role of DLC1 in cell migration

**Figure 2:**
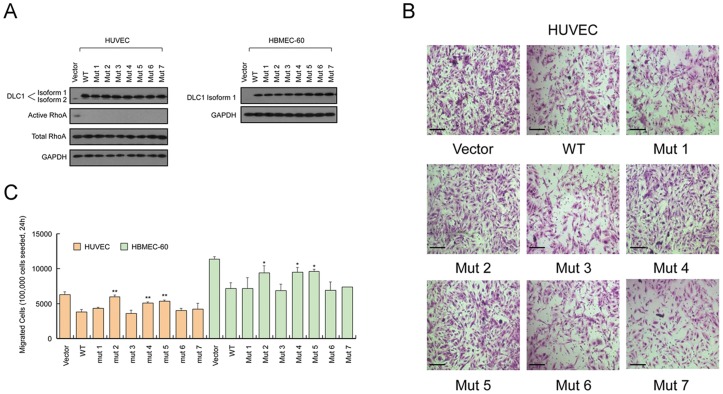
*DLC1* isoform 1 mutants had different effects on cell migration compared with the wild type protein. (A) Western blot analyses of *DLC1* isoform 1 mutant overexpression in two endothelial cell lines, HUVEC and HBMEC-60. In HUVECs, the effect of *DLC1* isoform 1 mutation on the GAP activity of the protein was detected by western blotting. (B) Representative images of the Transwell migration assay using HUVECs cells are shown. (C) The quantification of HUVEC and HBMEC-60 migration showed significant differences between wild-type DLC1 and Mutants 2, 4 and 5, whereas the other mutants showed no significant difference from wild-type DLC1. Wild-type DLC1 also showed an inhibitory effect on cell migration compared to the control vector. *Student's t-test 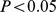; ** 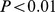. Scale bars, 100 µm. Ns, not significant.

To study whether the rare variants identified in the CHD cohort affect the protein function of DLC1, we cloned 7 of the variants, including 4 private variants and 3 other rare variants, by introducing the point mutations into the wild-type *DLC1* isoform 1. These variants are as the following: Mutant 1, Ala350Thr; Mutant 2, Met360Lys; Mutant 3, Leu413Met; Mutant 4, Glu418Lys; Mutant 5, Asp554Val; Mutant 6, Leu952Val; and Mutant 7, Val1371Leu. These seven variants were selected because they were absent in 900 control samples (altogether 10 rare variants were absent in 900 control samples, but mutant vectors of Gly266Glu, Thr433Asn and Ile1511Met were failed to construct for technical reasons). Cell migration inhibition is one of the most studied functions of DLC1. However, most studies focused on the isoform 2 of DLC1 (1091 aa) and the effect of isoform 1 and its mutants on cell migration has not been reported. Therefore, we assessed the functions of DLC1 isoform 1 and its mutants on migration in human umbilical vein endothelial cells (HUVEC) and human bone marrow endothelial cells 60 (HBMEC-60), the two cell lines widely used in cardiovascular disease studies. The wild-type isoform 1, mutants 1–7, and the control vector were transfected into HUVEC and HBMEC-60 cells (Fig. 2A), following by transwell migration assays to analyze the migratory abilities of the cells. As shown in Figure 2, DLC1 isoform 1 suppressed the migration abilities of HUVEC and HBMEC-60 *in vitro*. Mutants 2, 4 and 5 (Fig. 1D), which either changed the polarity (Met360 and Asp554) or altered the electric charge (Glu418) of the amino acids, rescued the migration suppression by the wild-type DLC1 protein, as the migration of the cells transfected by these mutants was similar to the control cells. The other mutants appeared to have no significant differences from the wild type to suppress cell migration (Fig. 2B, 2C). In addition, the migration rescue effect of Mutants 2, 4 and 5 could not be accounted for by their effect on cell proliferation, because the mutants and the wild-type protein similarly suppressed the growth of endothelial cells (Fig. S1 in File S1).

### The Glu418Lys mutant changes subcellular localization of DLC1

**Figure 3:**
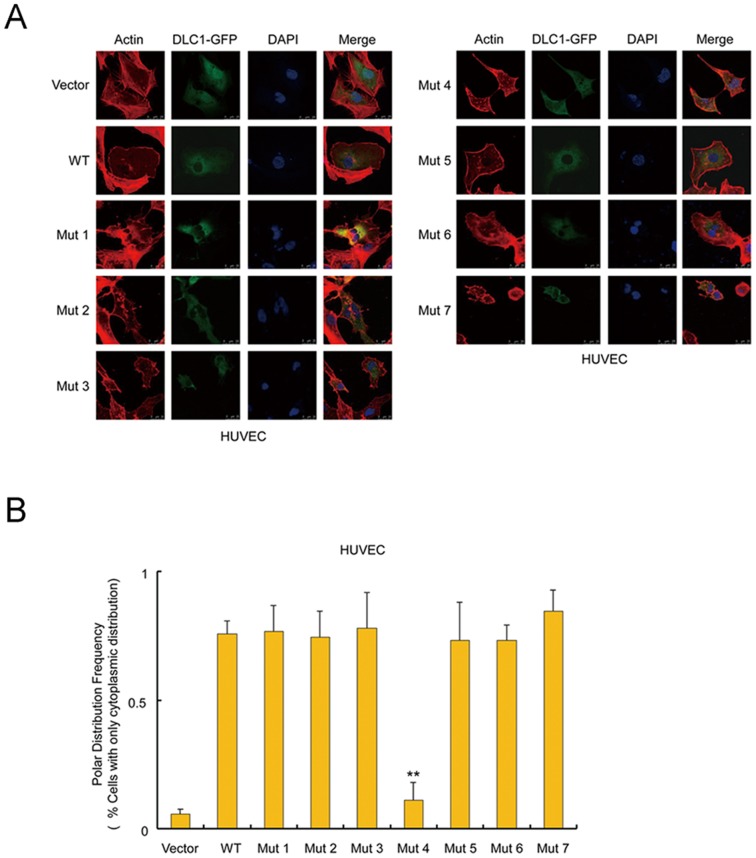
The subcellular localization of wild-type DLC1 and mutants in HUVECs. (A) Images of wild-type and mutant DLC1 distribution in HUVECs using laser scanning confocal microscopy. (B) The percentage of cells with wild-type and mutant DLC1 proteins with exclusive cytoplasmic-localization. Mutant 4 showed a significant difference from the wild type, as opposed to the control vector. *Student's t-test 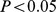; ** 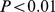. Scale bar, 25 µm. Ns, not significant.

DLC1 is an inhibitor protein of small GTPases including RhoA/B/C and CDC42. Such an inhibitory effect was thought to be mainly mediated by the GAP domain of DLC1. Interestingly, none of the variants identified in CHD lay within the GAP domain. Since a recent study reported that the protein sequences outside of GAP domain may also affect the Rho-inhibiting activity of DLC1 [40], we studied whether the CHD variants affect the GAP activity of DLC1. It was found all the mutants and the wild-type protein efficiently suppressed the activation of RhoA (Fig. 2A). Then we considered whether the small GTPases in the endothelial cells were regulated by DLC1 *in situ* by analyzing the formation of stress fibers in the cells, a process that is regulated by Rho activities. The *DLC1* constructs were tagged with GFP, and the stress fiber formation was analyzed by the high-affinity F-actin probe Rhodamine phalloidin. The data showed that when the wild-type and mutant DLC1 were expressed in the endothelial cells, the formation of stress fibers were prevented to similar levels (Fig. 3A, Fig. S3 in File S1).

Although the variants in DLC1 did not lead to any difference in the regulation of endothelial cytoskeleton, we observed Mutant 4 (Glu418Lys) markedly altered the localization of the protein in the cells. Fluorescent confocal microscopy revealed that DLC1 isoform 1 was primarily located in the cytoplasm, as were Mutants 1–3 and 5–7. Mutant 4 was found in both the cytoplasm and nucleus. Compared to the wild type and the other 6 mutant proteins which were excluded from the nucleus of 73% – 84% endothelial cells, the Mutant 4 protein was not seen in only 11% of the nucleus, suggesting the protein nuclear translocation (PNT) caused by the Glu418Lys substitution (Fig. 3). It was previously reported that PNT occurred in 10% of tumor cells after transfection with *DLC1* isoform 2 and was accompanied by morphological changes, and then these cells progressed to apoptosis stage [41]. Although no difference was observed between the cells transfected by Mutant 4 and those by other *DLC1* constructs in our apoptosis analysis, all the wild type and mutant DLC1 led to markedly enhanced percentages of apoptotic cells (Fig. S2 in File S1).

## Discussion

Congenital heart disease is complex. Although key mutations have been identified by pedigree research, the great heterogeneity of CHD makes it very difficult to identify the responsible genes, particularly among sporadic CHD cohorts. However, disease or deleterious alleles could be rare [42], and rare variants that have obvious functional consequences will show the largest effect size for the disease [43]. Therefore, we focused on the identification of rare variants in a case cohort. We successfully identified 13 rare variants in a sporadic CHD cohort and provide clear evidence that 8 rare variants are clustered in the N-terminal region of the protein. However, we should note that, the reference variant data from the 1000 Genomes Project and the Exome Sequencing Project were produced by different platforms, most of which were next generation sequencing platforms. The sequencing depth, coverage and data analysis pipelines might affect the variant detection rate. It is the consideration that the variant number from different platforms might not be compared directly. So we focused on the locations of the rare variants on the protein, and the analysis strategy is feasible in our study. More importantly, in our *in vitro* assays, three private variants (corresponding to Mutants 2, 4 and 5) were shown to alter the ability of DLC1 to inhibit cell migration or the subcellular localization of the protein, which supported the notion that private variants might also play major roles in the pathological process of complex diseases [43]. In addition, the extended N-terminal region of DLC1 isoform 1 harbors 83% (5/6) of the private variants identified in the CHD cohort in a non-random manner. The relatively high transcriptional level of *DLC1* isoform 1 in human heart tissues [28] implies that the unique N-terminal region may possess a tissue-specific function in the cardiovascular system. However, future studies are necessary to elucidate the details.

Cell migration is an evolutionarily conserved mechanism that includes four steps: polarization, protrusion, adhesion and retraction [44]. Actin is primarily involved in the last three steps. Studies have confirmed that DLC1 can function in the regulation of actin cytoskeletal organization and cell migration [45], suggesting that DLC1 acts as an important regulator of migration. It is essential for endothelial cells in the outflow tract (OT) and atrioventricular (AV) regions to migrate into the cardiac jelly during embryonic heart development [46]. Similarly, the migration of cardiac neural crest cells is also a crucial event during heart development, and the inappropriate timing or path of cardiac neural crest cell migration will cause cardiac congenital anomalies [47]. Thus, if the migration regulatory ability of DLC1 is impaired in the early stage of fetal cardiac development, it is reasonable to speculate that inaccurate developmental consequences, such as defects or malformations, will occur. Although DLC1 is generally considered to affect cell motility and focal adhesion via the Rho-Gap domain and focal adhesion targeting region, respectively [38], [39], [45], the SAM domain has also been reported to regulate cell migration [48]. We demonstrated that three private variants near the SAM domain could reduce the inhibitory effect of wild-type DLC1, suggesting that these mutations might be implicated in regulating the function of the SAM domain.

Although *DLC1* isoform 2 has been well studied during the past ten years, the functions of *DLC1* isoform 1 still need to be characterized. A series of assays were performed to verify whether DLC1 isoform 1 had a function similar to isoform 2. As shown above, all the mutant and wild-type protein had suppression effects on Rho (Fig. 2A), and similarly regulated the cytoskeleton rearrangement and prevented the formation of stress fiber in the endothelial cells (Fig. 3A, Fig. S3 in File S1). Considering that endocardium formation in the primitive heart tube is affected by vasculogenesis [49], we conducted an angiogenesis assay *in vitro*, and DLC1 isoform 1 and the mutants had similar prohibitive effects on angiogenesis (Fig. S4 in File S1). Although the mutants showed no difference from the wild-type protein, these negative results only indicate that the variations did not affect these specific features in certain cells. Indeed, the variants might impair the function of DLC1 in other ways or in other cardiac cells. Furthermore, to the best of our knowledge, this is the first report using in vitro assays to demonstrate that DLC1 isoform 1 manifests a function analogous to isoform 2. In conclusion, our mutational analysis of *DLC1* isoform 1 presents a spectrum of rare variants in a CHD cohort and shows a mutation cluster in the N-terminus of the DLC1 protein. Our functional assays prove that the ability to inhibit cell migration or the subcellular localization of the protein are altered by three private variants. These findings provide novel insight that *DLC1* may be a high-priority candidate gene associated with CHD.

## Supporting Information

File S1 **Tables S1–S4 and Figures S1–S4.** Table S1. The statistics of phenotype information of 148 non-trisomy CHD patients; Table S2. The primers for PCR to amplify the exons and portions of 5′UTR and 3′UTR regions of *DLC1* isoform 1; Table S3. Rare variants of *DLC1* isoform 1 identified in The 1000 Genomes project and Exome sequencing project; Table S4. The effects of 13 rare variants identified in the CHD cohort were predicted using multiple prediction algorithms; Figure S1. Effect of wild-type DLC1 isoform 1 and mutants on HUVEC proliferation; Figure S2. The apoptosis analysis of wild-type DLC1 isoform 1 and mutants in HUVECs; Figure S3. Percentage of cells overexpressing wild-type DLC1 isoform 1 and mutants that exhibited stress fibers; Figure S4. Wild-type DLC1 isoform 1 and mutants had similar effects on angiogenesis.
(DOC)
