# Supplementary material for: Uncovering the Rare Variants of DLC1 Isoform 1 and Their Functional Effects in a Chinese Sporadic Congenital Heart Disease Cohort
Source: PLoS One. 2014 Feb 28;9(2):e90215. doi: 10.1371/journal.pone.0090215 (PMC3938602; doi:10.1371/journal.pone.0090215)
Supplement: File S1 — Tables S1–S4 and Figures S1–S4. Table S1. The statistics of phenotype information of 148 non-trisomy CHD patients; Table S2. The primers for PCR to amplify the exons and portions of 5′UTR and 3′UTR regions of DLC1 isoform 1; Table S3. Rare variants of DLC1 isoform 1 identified in The 1000 Genomes project and Exome sequencing project; Table S4. The effects of 13 rare variants identified in the CHD cohort were predicted using multiple prediction algorithms; Figure S1. Effect of wild-type DLC1 isoform 1 and mutants on HUVEC proliferation; Figure S2. The apoptosis analysis of wild-type DLC1 isoform 1 and mutants in HUVECs; Figure S3. Percentage of cells overexpressing wild-type DLC1 isoform 1 and mutants that exhibited stress fibers; Figure S4. Wild-type DLC1 isoform 1 and mutants had similar effects on angiogenesis. (DOC) [file pone.0090215.s001.doc]

**Supplementary Tables**

**Table S1.** The statistics of phenotype information of 148 non-trisomy CHD patients.

| Item | Subtype | Number of cases |
| --- | --- | --- |
| Single subtype | VSD | 48 |
|  | PDA | 27 |
|  | ASD | 16 |
|  | TOF | 6 |
|  | PS | 4 |
|  | AVS | 2 |
|  | AI | 1 |
|  | AW | 1 |
|  | DORV | 1 |
|  | IAA | 1 |
| Combined subtypes^a^ | Including septal defects | 33 |
|  | Not including septal defects | 8 |

Notes. VSD, ventricular septal defect; PDA, patent ductus arteriosus; ASD, atrial septal defect; TOF, tetralogy of fallot; PS, pulmonary stenosis; AVS, aortic valve stenosis; AI, aortic insufficiency; AW, aorticopulmonary window; DORV, double outlet right ventricle; IAA, interrupted aortic arch. a, Combined subtypes indicate a patient carries more than one CHD subtypes (e.g., a patient with VSD, patent foramen ovale (PFO) and pulmonary hypertension (PH)).

**Table S2.** The primers for PCR to amplify the exons and portions of 5’UTR and 3’UTR regions of *DLC1* isoform 1.

| ID | Sequence (5' to 3') |
| --- | --- |
| DLC1-Exon1-F | CTCTACAGCTAATCACC |
| DLC1-Exon1-R | TATGGCTTTGCTCTAA |
| DLC1-Exon2-1-F | TTAACTTTGATAGCATAG |
| DLC1-Exon2-1-R | ATCAGCATTGTTATCC |
| DLC1-Exon2-2-F | GCAGGCAAGTATGGAAA |
| DLC1-Exon2-2-R | TACAGCAGAGTTAAGCA |
| DLC1-Exon2-3-F | GTAAAAGAAAGTGGGGAGA |
| DLC1-Exon2-3-R | ATAACAGGGCAAAAGAACC |
| DLC1-Exon3-F | TAAAGACTGCCTATTATCC |
| DLC1-Exon3-R | TGGTACTGTACTGGGGTTA |
| DLC1-Exon4-F | TGGGAGAAAGAAGAAGTGA |
| DLC1-Exon4-R | TGGCTAAGATTCCAACAGT |
| DLC1-Exon5-F | CAAAGACAGCTCTAGTGAT |
| DLC1-Exon5-R | CTTACATATTGAGCAAAAC |
| DLC1-Exon6-F | TAAGCGTGTATGTCTGT |
| DLC1-Exon6-R | GTGTTACCAAGGTGCT |
| DLC1-Exon7-F | CCCATAGAACTTGACGG |
| DLC1-Exon7-R | TTAGCAAGAACAAAAGC |
| DLC1-Exon8-F | TTAGCCAGGATGGTCTC |
| DLC1-Exon8-R | TGCTTACGGAACAACAA |
| DLC1-Exon9-1-F | CAGCCTGGGCAATACA |
| DLC1-Exon9-1-R | TCCATCCGTTTCAGCA |
| DLC1-Exon9-2-F | CGTCATCAGCGTTTGC |
| DLC1-Exon9-2-R | CACAGAGCCGTTATTCC |
| DLC1-Exon9-3-F | ACGTGGTGGAGCAGAA |
| DLC1-Exon9-3-R | GGAATGGACATGGCTAA |
| DLC1-Exon10-F | TGGACAACATAGGGAGAC |
| DLC1-Exon10-R | TTGCCATAGGATGACAGAT |
| DLC1-Exon11-12-F | GGAGCCCACAGGGTTTGC |
| DLC1-Exon11-12-R | GGATGACAGAGCGAGACT |
| DLC1-Exon13-F | CTGGGCTGAGGTATTTGA |
| DLC1-Exon13-R | ATGTCTTTATTAGCGGTGT |
| DLC1-Exon14-F | TCTGGTGACCAAAGTAAAA |
| DLC1-Exon14-R | AAGACATTCTCAGGCTATT |
| DLC1-Exon15-F | TGTTCCTACCCAGTCT |
| DLC1-Exon15-R | ATTAGTTGGCAAAAGC |
| DLC1-Exon16-F | CTTGGGGACAGCAGCCTATT |
| DLC1-Exon16-R | TATGCCCGGCCTCTACTTTA |
| DLC1-Exon17-F | AGGATGCCATTTACCT |
| DLC1-Exon17-R | GTCGCTGAAAGACCAA |
| DLC1-Exon18-F | CGACCACTTCGTAATT |
| DLC1-Exon18-R | GGATACACCAGTCCCT |

**Table S3.** Rare variants of *DLC1* isoform 1 identified in The 1000 Genomes project and Exome sequencing project.

| **Source** | **rs ID** | **Chromosome** | **Position (Hg19)** | **Reference Base** | **Sample Alleles** | **Amino Acids** | **Protein Position** | **SIFT Prediction (**$\mathbf{cutoff}=\mathbf{0}.\mathbf{05}$**)** |
| --- | --- | --- | --- | --- | --- | --- | --- | --- |
| 1000 Genomes | rs78322853 | 8 | 12943374 | G | C/G | ILE>MET | 1511 | Damaging |
| 1000 Genomes | rs142253103 | 8 | 12943382 | C | C/T | VAL>ILE | 1509 | Tolerated |
| 1000 Genomes/ESP | Na | 8 | 12943417 | G | A/G | THR>ILE | 1497 | Damaging |
| 1000 Genomes | Na | 8 | 12943855 | A | A/C | ILE>MET | 1470 | Tolerated |
| 1000 Genomes/ESP | Na | 8 | 12943905 | C | C/T | ALA>THR | 1454 | Tolerated |
| 1000 Genomes | Na | 8 | 12943907 | C | C/T | ARG>HIS | 1453 | Damaging |
| 1000 Genomes/ESP | Na | 8 | 12943937 | G | C/G | ALA>GLY | 1443 | Tolerated |
| 1000 Genomes/ESP | rs146462979 | 8 | 12943944 | C | C/T | ALA>THR | 1441 | Tolerated |
| 1000 Genomes/ESP | Na | 8 | 12946014 | C | C/T | ARG>GLN | 1425 | Damaging |
| 1000 Genomes | Na | 8 | 12946029 | G | C/G | ALA>GLY | 1420 | Tolerated |
| 1000 Genomes | Na | 8 | 12946147 | T | C/T | ILE>VAL | 1381 | Tolerated |
| 1000 Genomes | rs142865083 | 8 | 12946177 | C | C/G | VAL>LEU | 1371 | Damaging |
| 1000 Genomes | rs1127609 | 8 | 12946197 | G | C/G | PRO>ARG | 1364 | Damaging |
| 1000 Genomes | Na | 8 | 12947768 | T | C/T | TYR>CYS | 1356 | Tolerated |
| 1000 Genomes/ESP | Na | 8 | 12947771 | G | C/G | SER>CYS | 1355 | Damaging |
| 1000 Genomes/ESP | rs139251311 | 8 | 12947789 | G | A/G | SER>LEU | 1349 | Tolerated |
| 1000 Genomes/ESP | rs61752025 | 8 | 12947856 | C | A/C | ASP>TYR | 1327 | Damaging |
| 1000 Genomes/ESP | Na | 8 | 12947866 | G | C/G | HIS>GLN | 1323 | Tolerated |
| 1000 Genomes | Na | 8 | 12947875 | G | G/T | ASP>GLU | 1320 | Tolerated |
| 1000 Genomes | rs146094242 | 8 | 12947888 | T | C/T | ASP>GLY | 1316 | Tolerated |
| 1000 Genomes/ESP | rs142572997 | 8 | 12947898 | G | C/G | LEU>VAL | 1313 | Tolerated |
| 1000 Genomes | rs146713932 | 8 | 12947900 | T | C/T | HIS>ARG | 1312 | Tolerated |
| 1000 Genomes/ESP | rs143071249 | 8 | 12947907 | G | C/G | LEU>VAL | 1310 | Damaging |
| 1000 Genomes/ESP | rs150404537 | 8 | 12947918 | G | C/G | THR>SER | 1306 | Tolerated |
| 1000 Genomes | Na | 8 | 12947932 | C | C/G | GLU>ASP | 1301 | Tolerated |
| 1000 Genomes/ESP | Na | 8 | 12947954 | C | C/T | ARG>HIS | 1294 | Tolerated |
| 1000 Genomes/ESP | rs200504821 | 8 | 12947960 | C | C/T | ARG>GLN | 1292 | Tolerated |
| 1000 Genomes/ESP | Na | 8 | 12947973 | C | C/T | GLU>LYS | 1288 | Tolerated |
| ESP | Na | 8 | 12948877 | CAGCT | C | Frame Shift |  | Na |
| ESP | Na | 8 | 12948878 | A | AG | Frame Shift |  | Na |
| 1000 Genomes | Na | 8 | 12948888 | T | C/T | GLU>GLY | 1265 | Damaging |
| 1000 Genomes | Na | 8 | 12948917 | C | C/G | LEU>PHE | 1255 | Damaging |
| 1000 Genomes/ESP | rs138181048 | 8 | 12948940 | C | C/T | VAL>ILE | 1248 | Tolerated |
| 1000 Genomes/ESP | rs200392347 | 8 | 12950166 | G | C/G | SER>CYS | 1232 | Damaging |
| 1000 Genomes | Na | 8 | 12950182 | C | C/T | VAL>MET | 1227 | Damaging |
| 1000 Genomes | rs142779460 | 8 | 12950194 | T | G/T | THR>PRO | 1223 | Damaging |
| 1000 Genomes | Na | 8 | 12950217 | A | A/T | VAL>GLU | 1215 | Damaging |
| 1000 Genomes/ESP | Na | 8 | 12950218 | C | C/T | VAL>ILE | 1215 | Damaging |
| 1000 Genomes | Na | 8 | 12950233 | C | C/T | ASP>ASN | 1210 | Damaging |
| 1000 Genomes/ESP | Na | 8 | 12950235 | C | C/T | SER>ASN | 1209 | Tolerated |
| 1000 Genomes | rs1044094 | 8 | 12950236 | T | A/T | SER>CYS | 1209 | Damaging |
| 1000 Genomes | Na | 8 | 12950248 | G | A/G | LEU>PHE | 1205 | Damaging |
| 1000 Genomes | rs1044093 | 8 | 12950265 | T | A/T | GLU>VAL | 1199 | Damaging |
| 1000 Genomes/ESP | Na | 8 | 12950275 | C | C/T | GLU>LYS | 1196 | Damaging |
| 1000 Genomes/ESP | rs146051142 | 8 | 12950310 | T | A/T | GLN>LEU | 1184 | Damaging |
| 1000 Genomes/ESP | Na | 8 | 12950317 | G | A/G | ARG>CYS | 1182 | Damaging |
| 1000 Genomes | Na | 8 | 12952285 | A | A/G | PHE>SER | 1170 | Damaging |
| 1000 Genomes/ESP | Na | 8 | 12952301 | T | G/T | LYS>GLN | 1165 | Damaging |
| 1000 Genomes/ESP | rs144835895 | 8 | 12952345 | A | A/T | LEU>GLN | 1150 | Damaging |
| 1000 Genomes/ESP | Na | 8 | 12952346 | G | C/G | LEU>VAL | 1150 | Tolerated |
| 1000 Genomes | Na | 8 | 12952379 | C | C/T | GLU>LYS | 1139 | Damaging |
| 1000 Genomes/ESP | Na | 8 | 12952383 | G | C/G | ASN>LYS | 1137 | Tolerated |
| 1000 Genomes/ESP | Na | 8 | 12952393 | T | C/T | ASP>GLY | 1134 | Tolerated |
| 1000 Genomes | Na | 8 | 12952397 | T | C/T | ILE>VAL | 1133 | Tolerated |
| 1000 Genomes/ESP | rs147918530 | 8 | 12952432 | C | C/T | ARG>GLN | 1121 | Damaging |
| 1000 Genomes | rs1127608 | 8 | 12952453 | C | C/T | ARG>LYS | 1114 | Damaging |
| 1000 Genomes | Na | 8 | 12952600 | C | C/T | ASP>ASN | 1108 | Damaging |
| 1000 Genomes/ESP | Na | 8 | 12952609 | G | A/G | HIS>TYR | 1105 | Damaging |
| 1000 Genomes | Na | 8 | 12952639 | T | C/T | ILE>VAL | 1095 | Tolerated |
| 1000 Genomes/ESP | rs140952951 | 8 | 12952684 | G | C/G | LEU>VAL | 1080 | Damaging |
| 1000 Genomes/ESP | rs150186144 | 8 | 12952750 | C | C/T | VAL>MET | 1058 | Tolerated |
| 1000 Genomes/ESP | Na | 8 | 12955933 | G | C/G | PRO>ALA | 1048 | Tolerated |
| 1000 Genomes/ESP | rs146001186 | 8 | 12956017 | T | A/T | ILE>PHE | 1020 | Damaging |
| 1000 Genomes/ESP | rs138719404 | 8 | 12956038 | G | C/G | LEU>VAL | 1013 | Tolerated |
| 1000 Genomes/ESP | rs201145872 | 8 | 12956046 | C | C/T | ARG>GLN | 1010 | Tolerated |
| 1000 Genomes/ESP | rs149295187 | 8 | 12956081 | G | C/G | HIS>GLN | 998 | Tolerated |
| 1000 Genomes | Na | 8 | 12956961 | C | C/G | SER>THR | 962 | Damaging |
| 1000 Genomes/ESP | Na | 8 | 12956967 | G | A/G | THR>ILE | 960 | Tolerated |
| 1000 Genomes/ESP | rs148423187 | 8 | 12956970 | G | C/G | THR>SER | 959 | Tolerated |
| 1000 Genomes | rs34336066 | 8 | 12956969-12956970 | GG | GGG | Frame Shift |  | Na |
| 1000 Genomes | rs121908500 | 8 | 12956971 | T | C/T | THR>ALA | 959 | Tolerated |
| 1000 Genomes | Na | 8 | 12956992 | G | C/G | LEU>VAL | 952 | Damaging |
| ESP | Na | 8 | 12956993 | GTGTATC | G | ILE GLU Lost |  | Tolerated |
| 1000 Genomes/ESP | rs142520789 | 8 | 12956993 | G | C/G | HIS>GLN | 951 | Tolerated |
| 1000 Genomes/ESP | rs147130694 | 8 | 12956995 | G | A/G | HIS>TYR | 951 | Tolerated |
| 1000 Genomes/ESP | rs141028457 | 8 | 12957015 | G | A/G | PRO>LEU | 944 | Damaging |
| 1000 Genomes/ESP | Na | 8 | 12957030 | G | A/G | SER>LEU | 939 | Tolerated |
| 1000 Genomes | Na | 8 | 12957156 | A | A/G | LEU>PRO | 897 | Tolerated |
| 1000 Genomes | Na | 8 | 12957181 | A | A/G | SER>PRO | 889 | Tolerated |
| 1000 Genomes/ESP | rs140570414 | 8 | 12957187 | G | C/G | LEU>VAL | 887 | Damaging |
| 1000 Genomes | Na | 8 | 12957214 | T | C/T | ILE>VAL | 878 | Tolerated |
| 1000 Genomes/ESP | rs145263649 | 8 | 12957234 | G | C/G | SER>CYS | 871 | Damaging |
| 1000 Genomes/ESP | Na | 8 | 12957251 | T | G/T | ARG>SER | 865 | Damaging |
| 1000 Genomes/ESP | rs149156083 | 8 | 12957264 | T | A/T | LYS>MET | 861 | Damaging |
| 1000 Genomes/ESP | Na | 8 | 12957270 | C | C/T | SER>ASN | 859 | Tolerated |
| 1000 Genomes | Na | 8 | 12957280 | T | C/T | SER>GLY | 856 | Tolerated |
| 1000 Genomes/ESP | Na | 8 | 12957314 | G | C/G | HIS>GLN | 844 | Damaging |
| 1000 Genomes/ESP | Na | 8 | 12957375 | T | C/T | ASN>SER | 824 | Tolerated |
| 1000 Genomes/ESP | Na | 8 | 12957385 | C | C/T | ALA>THR | 821 | Damaging |
| 1000 Genomes/ESP | Na | 8 | 12957387 | T | C/T | LYS>ARG | 820 | Tolerated |
| 1000 Genomes | Na | 8 | 12957399 | C | C/G | GLY>ALA | 816 | Damaging |
| 1000 Genomes | Na | 8 | 12957424 | A | A/G | TYR>HIS | 808 | Tolerated |
| 1000 Genomes/ESP | Na | 8 | 12957468 | T | C/T | GLN>ARG | 793 | Tolerated |
| 1000 Genomes/ESP | Na | 8 | 12957497 | A | A/C | ASN>LYS | 783 | Tolerated |
| 1000 Genomes | rs139243841 | 8 | 12957498 | T | C/T | ASN>SER | 783 | Tolerated |
| 1000 Genomes/ESP | Na | 8 | 12957522 | T | G/T | TYR>SER | 775 | Damaging |
| 1000 Genomes | Na | 8 | 12957537 | T | C/T | LYS>ARG | 770 | Damaging |
| 1000 Genomes/ESP | rs144923726 | 8 | 12957567 | G | A/G | THR>MET | 760 | Damaging |
| 1000 Genomes | Na | 8 | 12957582 | G | A/G | THR>MET | 755 | Damaging |
| 1000 Genomes | rs112183774 | 8 | 12957594 | C | C/T | SER>ASN | 751 | Damaging |
| ESP | Na | 8 | 12957610 | GGCTGCT | G | 2 SER Lost |  | Na |
| 1000 Genomes/ESP | rs149472569 | 8 | 12957633 | G | A/G | THR>MET | 738 | Damaging |
| 1000 Genomes/ESP | Na | 8 | 12957639 | T | C/T | ASN>SER | 736 | Tolerated |
| 1000 Genomes/ESP | rs140340878 | 8 | 12957657 | C | C/T | ARG>GLN | 730 | Tolerated |
| 1000 Genomes/ESP | rs142691117 | 8 | 12957678 | C | C/T | ARG>HIS | 723 | Tolerated |
| 1000 Genomes/ESP | rs199759496 | 8 | 12957696 | G | C/G | SER>CYS | 717 | Damaging |
| 1000 Genomes/ESP | Na | 8 | 12957698 | G | C/G | ILE>MET | 716 | Damaging |
| 1000 Genomes/ESP | rs114637912 | 8 | 12957699 | A | A/G | ILE>THR | 716 | Damaging |
| 1000 Genomes | rs1044092 | 8 | 12957711 | T | C/T | ASN>SER | 712 | Tolerated |
| 1000 Genomes/ESP | Na | 8 | 12957718 | G | C/G | GLN>GLU | 710 | Tolerated |
| 1000 Genomes/ESP | Na | 8 | 12957754 | T | C/T | ILE>VAL | 698 | Tolerated |
| 1000 Genomes/ESP | rs142931995 | 8 | 12957789 | G | C/G | ALA>GLY | 686 | Tolerated |
| 1000 Genomes/ESP | Na | 8 | 12957805 | G | A/G | HIS>TYR | 681 | Damaging |
| 1000 Genomes/ESP | rs139778805 | 8 | 12957850 | T | A/T | SER>CYS | 666 | Damaging |
| 1000 Genomes/ESP | Na | 8 | 12957852 | C | C/T | ARG>HIS | 665 | Damaging |
| 1000 Genomes | rs143161954 | 8 | 12957889 | T | C/T | MET>VAL | 653 | Tolerated |
| 1000 Genomes/ESP | Na | 8 | 12957891 | C | A/C | SER>ILE | 652 | Tolerated |
| 1000 Genomes | rs146812798 | 8 | 12957909 | A | A/C | LEU>ARG | 646 | Damaging |
| 1000 Genomes/ESP | rs200394870 | 8 | 12957946 | C | A/C | ASP>TYR | 634 | Damaging |
| 1000 Genomes/ESP | rs145626397 | 8 | 12957969 | G | A/G | SER>PHE | 626 | Damaging |
| 1000 Genomes/ESP | Na | 8 | 12957996 | C | C/G | ARG>PRO | 617 | Damaging |
| 1000 Genomes | rs148893339 | 8 | 12958020 | G | C/G | PRO>ARG | 609 | Tolerated |
| 1000 Genomes/ESP | Na | 8 | 12958024 | G | A/G | PRO>SER | 608 | Tolerated |
| 1000 Genomes | rs148021240 | 8 | 12958048 | T | G/T | THR>PRO | 600 | Tolerated |
| 1000 Genomes | Na | 8 | 12958050 | C | C/T | SER>ASN | 599 | Damaging |
| 1000 Genomes/ESP | Na | 8 | 12958075 | C | A/C | VAL>LEU | 591 | Tolerated |
| 1000 Genomes | Na | 8 | 12958111 | C | C/T | GLY>ARG | 579 | Damaging |
| 1000 Genomes/ESP | rs145901252 | 8 | 12958116 | C | C/T | SER>ASN | 577 | Damaging |
| 1000 Genomes/ESP | Na | 8 | 12958131 | G | A/G | PRO>LEU | 572 | Tolerated |
| 1000 Genomes/ESP | rs139415835 | 8 | 12958146 | G | A/G | PRO>LEU | 567 | Damaging |
| 1000 Genomes | rs147537968 | 8 | 12958147 | G | C/G | PRO>ALA | 567 | Damaging |
| 1000 Genomes/ESP | rs149628772 | 8 | 12958149 | G | G/T | SER>TYR | 566 | Tolerated |
| 1000 Genomes | Na | 8 | 12958163 | G | G/T | ASP>GLU | 561 | Tolerated |
| 1000 Genomes/ESP | Na | 8 | 12958165 | C | C/T | ASP>ASN | 561 | Tolerated |
| 1000 Genomes/ESP | Na | 8 | 12958219 | C | A/C | ASP>TYR | 543 | Damaging |
| 1000 Genomes/ESP | rs148781138 | 8 | 12968266 | A | A/G | ILE>THR | 496 | Damaging |
| 1000 Genomes/ESP | Na | 8 | 12968294 | C | C/G | GLU>GLN | 487 | Damaging |
| 1000 Genomes/ESP | Na | 8 | 12968305 | A | A/G | LEU>SER | 483 | Tolerated |
| 1000 Genomes/ESP | rs200557469 | 8 | 12968315 | C | C/T | ASP>ASN | 480 | Tolerated |
| 1000 Genomes/ESP | rs146824981 | 8 | 12973112 | T | A/T | TYR>PHE | 468 | Damaging |
| 1000 Genomes | Na | 8 | 12973148 | G | A/G | ALA>VAL | 456 | Damaging |
| 1000 Genomes | Na | 8 | 12973149 | C | C/T | ALA>THR | 456 | Damaging |
| 1000 Genomes/ESP | rs200549738 | 8 | 13162780 | G | G/T | ALA>ASP | 449 | Damaging |
| 1000 Genomes/ESP | Na | 8 | 13162808 | T | A/T | ILE>PHE | 440 | Tolerated |
| 1000 Genomes | rs7005578 | 8 | 13251067 | T | C/T | THR>ALA | 437 | Tolerated |
| 1000 Genomes/ESP | Na | 8 | 13251087 | T | C/T | ASN>SER | 430 | Tolerated |
| 1000 Genomes | Na | 8 | 13251111 | A | A/G | LEU>PRO | 422 | Damaging |
| 1000 Genomes/ESP | rs145921105 | 8 | 13251115 | C | C/T | ASP>ASN | 421 | Tolerated |
| 1000 Genomes/ESP | rs73551600 | 8 | 13251126 | G | A/G | THR>ILE | 417 | Tolerated |
| 1000 Genomes/ESP | Na | 8 | 13251128 | G | G/T | ASP>GLU | 416 | Damaging |
| 1000 Genomes/ESP | rs143447199 | 8 | 13251139 | A | A/T | LEU>MET | 413 | Damaging |
| 1000 Genomes/ESP | Na | 8 | 13251147 | C | C/T | ARG>GLN | 410 | Tolerated |
| 1000 Genomes/ESP | Na | 8 | 13251165 | A | A/G | ILE>THR | 404 | Tolerated |
| 1000 Genomes | Na | 8 | 13251193 | A | A/C | SER>ALA | 395 | Tolerated |
| 1000 Genomes/ESP | rs141624794 | 8 | 13251201 | T | A/T | ASP>VAL | 392 | Damaging |
| 1000 Genomes/ESP | rs150701452 | 8 | 13258989 | C | C/T | ARG>GLN | 388 | Tolerated |
| 1000 Genomes/ESP | rs143448922 | 8 | 13258990 | G | A/G | ARG>TRP | 388 | Damaging |
| 1000 Genomes | rs142165160 | 8 | 13258992 | C | C/T | ARG>GLN | 387 | Tolerated |
| 1000 Genomes/ESP | rs139628612 | 8 | 13259044 | C | C/T | GLU>LYS | 370 | Damaging |
| 1000 Genomes | rs147994291 | 8 | 13259074 | T | C/T | MET>VAL | 360 | Damaging |
| 1000 Genomes/ESP | rs143724114 | 8 | 13259100 | C | C/T | ARG>GLN | 351 | Damaging |
| 1000 Genomes/ESP | rs144283917 | 8 | 13259101 | G | A/G | ARG>TRP | 351 | Damaging |
| 1000 Genomes/ESP | rs148727515 | 8 | 13259103 | G | A/G | ALA>VAL | 350 | Damaging |
| 1000 Genomes | rs138797224 | 8 | 13259109 | T | G/T | ASP>ALA | 348 | Damaging |
| ESP | Na | 8 | 13259113 | G | A/G | ARG>stop | 347 | Na |
| 1000 Genomes/ESP | rs191904737 | 8 | 13356569 | G | A/G | ARG>CYS | 338 | Damaging |
| 1000 Genomes/ESP | rs142262550 | 8 | 13356572 | G | C/G | LEU>VAL | 337 | Tolerated |
| 1000 Genomes/ESP | rs151016316 | 8 | 13356607 | A | A/G | LEU>PRO | 325 | Tolerated |
| 1000 Genomes | rs111350657 | 8 | 13356650 | T | C/T | LYS>GLU | 311 | Tolerated |
| 1000 Genomes/ESP | rs141332017 | 8 | 13356678 | T | A/T | GLN>HIS | 301 | Damaging |
| 1000 Genomes/ESP | rs150334629 | 8 | 13356737 | G | A/G | PRO>SER | 282 | Tolerated |
| 1000 Genomes/ESP | rs143012126 | 8 | 13356764 | C | C/T | ASP>ASN | 273 | Tolerated |
| 1000 Genomes | rs111395470 | 8 | 13356794 | T | G/T | THR>PRO | 263 | Damaging |
| 1000 Genomes/ESP | rs146364788 | 8 | 13356810 | G | G/T | PHE>LEU | 257 | Tolerated |
| 1000 Genomes/ESP | rs146692656 | 8 | 13356812 | A | A/T | PHE>ILE | 257 | Tolerated |
| 1000 Genomes | Na | 8 | 13356829 | T | C/T | ASN>SER | 251 | Tolerated |
| 1000 Genomes/ESP | rs149776829 | 8 | 13356859 | G | G/T | PRO>HIS | 241 | Tolerated |
| 1000 Genomes/ESP | rs61757614 | 8 | 13356860 | G | C/G | PRO>ALA | 241 | Tolerated |
| 1000 Genomes/ESP | Na | 8 | 13356863 | G | G/T | PRO>THR | 240 | Tolerated |
| 1000 Genomes/ESP | rs140615088 | 8 | 13356877 | C | C/T | ARG>GLN | 235 | Tolerated |
| 1000 Genomes | rs75453454 | 8 | 13356914 | T | C/T | LYS>GLU | 223 | Tolerated |
| 1000 Genomes/ESP | rs144566531 | 8 | 13356962 | T | G/T | LYS>GLN | 207 | Tolerated |
| 1000 Genomes | Na | 8 | 13356967 | G | A/G | ALA>VAL | 205 | Tolerated |
| 1000 Genomes/ESP | Na | 8 | 13357005 | C | A/C | GLU>ASP | 192 | Damaging |
| 1000 Genomes/ESP | Na | 8 | 13357007 | C | C/T | GLU>LYS | 192 | Damaging |
| 1000 Genomes | rs145216060 | 8 | 13357042 | T | G/T | GLU>ALA | 180 | Damaging |
| 1000 Genomes | Na | 8 | 13357049 | T | A/T | SER>CYS | 178 | Damaging |
| 1000 Genomes/ESP | rs141370387 | 8 | 13357067 | A | A/C | LEU>VAL | 172 | Tolerated |
| 1000 Genomes | Na | 8 | 13357094 | A | A/C | SER>ALA | 163 | Damaging |
| 1000 Genomes/ESP | rs150963046 | 8 | 13357118 | G | G/T | GLN>LYS | 155 | Damaging |
| 1000 Genomes/ESP | Na | 8 | 13357186 | C | C/T | GLY>GLU | 132 | Tolerated |
| 1000 Genomes/ESP | rs140759185 | 8 | 13357234 | T | C/T | ASN>SER | 116 | Tolerated |
| 1000 Genomes | Na | 8 | 13357261 | A | A/T | LEU>GLN | 107 | Damaging |
| 1000 Genomes/ESP | rs150090193 | 8 | 13357316 | C | C/T | ASP>ASN | 89 | Damaging |
| 1000 Genomes/ESP | rs138468563 | 8 | 13357379 | A | A/T | SER>THR | 68 | Tolerated |
| 1000 Genomes/ESP | rs143324598 | 8 | 13357414 | T | C/T | GLU>GLY | 56 | Damaging |
| 1000 Genomes/ESP | Na | 8 | 13357420 | C | C/T | ARG>HIS | 54 | Tolerated |
| 1000 Genomes/ESP | Na | 8 | 13357421 | G | A/G | ARG>CYS | 54 | Damaging |
| 1000 Genomes | Na | 8 | 13357444 | T | C/T | LYS>ARG | 46 | Tolerated |
| 1000 Genomes/ESP | Na | 8 | 13357459 | T | C/T | GLN>ARG | 41 | Damaging |
| 1000 Genomes | Na | 8 | 13357464 | G | G/T | SER>ARG | 39 | Tolerated |
| 1000 Genomes | Na | 8 | 13357528 | A | A/T | MET>LYS | 18 | Damaging |
| 1000 Genomes/ESP | rs201093868 | 8 | 13357530 | C | C/G | TRP>CYS | 17 | Damaging |

**Table S4.** The effects of 13 rare variants identified in the CHD cohort were predicted using multiple prediction algorithms.

| **Patient ID** | **Diagnosis** | **Nucleotide alteration^a^** | **Amino acid alteration** | **PolyPhen-2 score (HumDiv)** | **PolyPhen-2 prediction (HumDiv)** | **LRT score** | **LRT prediction** | **Mutation Taster score** | **Mutation Taster prediction** | **Mutation Assessor score** | **Mutation Assessor prediction** | **PhyloP** | **SiPhy** |
| --- | --- | --- | --- | --- | --- | --- | --- | --- | --- | --- | --- | --- | --- |
| 67 | VSD&PFO | c.797G>A | p.Gly266Glu | 0.046 | Benign | 0.3538 | Neutral | 0.0016 | Polymorphism | 1.65 | low | 0.38 | 8.2798 |
| 153 | VSD | c.1048G>A* | p.Ala350Thr | 0.005 | Benign | 0.2680 | Neutral | 0.0000 | Polymorphism | 0.205 | neutral | 0.35 | 1.186 |
| 168 | ASD | c.1079T>A* | p.Met360Lys | 0.999 | Probably damaging | 0.0001 | Deleterious | 0.0002 | Polymorphism | 1.445 | low | 2.222 | 14.4619 |
| 169 | PS | c.1252G>A* | p.Glu418Lys | 0.873 | Possibly damaging | 0.7168 | Neutral | 0.0714 | Polymorphism | 1.04 | low | 2.658 | 12.0498 |
| 89 | PDA | c.1298C>A | p.Thr433Asn | 1 | Probably damaging | 0.0115 | Neutral | 0.9369 | Disease causing | 1.355 | low | 2.651 | 16.5171 |
| 131 | PDA | c.[1661A>T(+)1662T>C]* | p.Asp554Val | 0.974 | Probably damaging | 0.0002 | Deleterious | Na | Na | 1.83 | low | 2.324 | 15.5735 |
| 190 | VSD | c.[1661A>T(+)1662T>C]* | p.Asp554Val | 0.974 | Probably damaging | 0.0002 | Deleterious | Na | Na | 1.83 | low | 2.324 | 15.5735 |
| 49 | TOF | c.659C>T | p.Ala220Val | 0.001 | Benign | 0.1624 | Neutral | 0.0000 | Polymorphism | -0.205 | neutral | 0.102 | 5.4931 |
| 61 | TOF | c.1051C>T | p.Arg351Trp | 1 | Probably damaging | 0.0017 | Neutral | 0.0002 | Polymorphism | 1.445 | low | 1.413 | 14.1929 |
| 42 | VSD | c.1237T>A* | p.Leu413Met | 0.989 | Probably damaging | 0.4053 | Neutral | 0.0751 | Polymorphism | 1.355 | low | -1.629 | 3.8601 |
| 55 | PDA | c.1683C>A | p.Asp561Glu | 0.029 | Benign | 0.0975 | Neutral | 0.2364 | Polymorphism | 0.69 | neutral | 0.425 | 2.0255 |
| 124 | VSD | c.2854C>G* | p.Leu952Val | 1 | Probably damaging | 0.0000 | Deleterious | 0.9998 | Disease causing | 1.715 | low | 1.441 | 10.8622 |
| 28 | VSD | c.4111G>C* | p.Val1371Leu | 0.018 | Benign | 0.0002 | Deleterious | 0 | Polymorphism | 0.805 | low | 1.062 | 11.2128 |
| 8 | VSD | c.4533C>G | p.Ile1511Met | 1 | Probably damaging | 0 | Deleterious | 1.0000 | Disease causing | 2.54 | medium | 0.451 | 6.571 |

Note. The predictions were done by ANNOVAR [1]. Na, no available data; VSD, ventricular septal defect; PFO, patent foramen ovale; ASD, atrial septal defect; PS, pulmonary stenosis; PDA, patent ductus arteriosus; TOF, tetralogy of Fallot. a, Nucleotide numbering is according to the RefSeq database NM_182643.2. *The mutant vectors were constructed according to these variants.

**Supplemental References**

1. Wang K, Li M and Hakonarson H (2010) ANNOVAR: functional annotation of genetic variants from high-throughput sequencing data. Nucleic Acids Res 38: e164.

**Supplementary Figures**


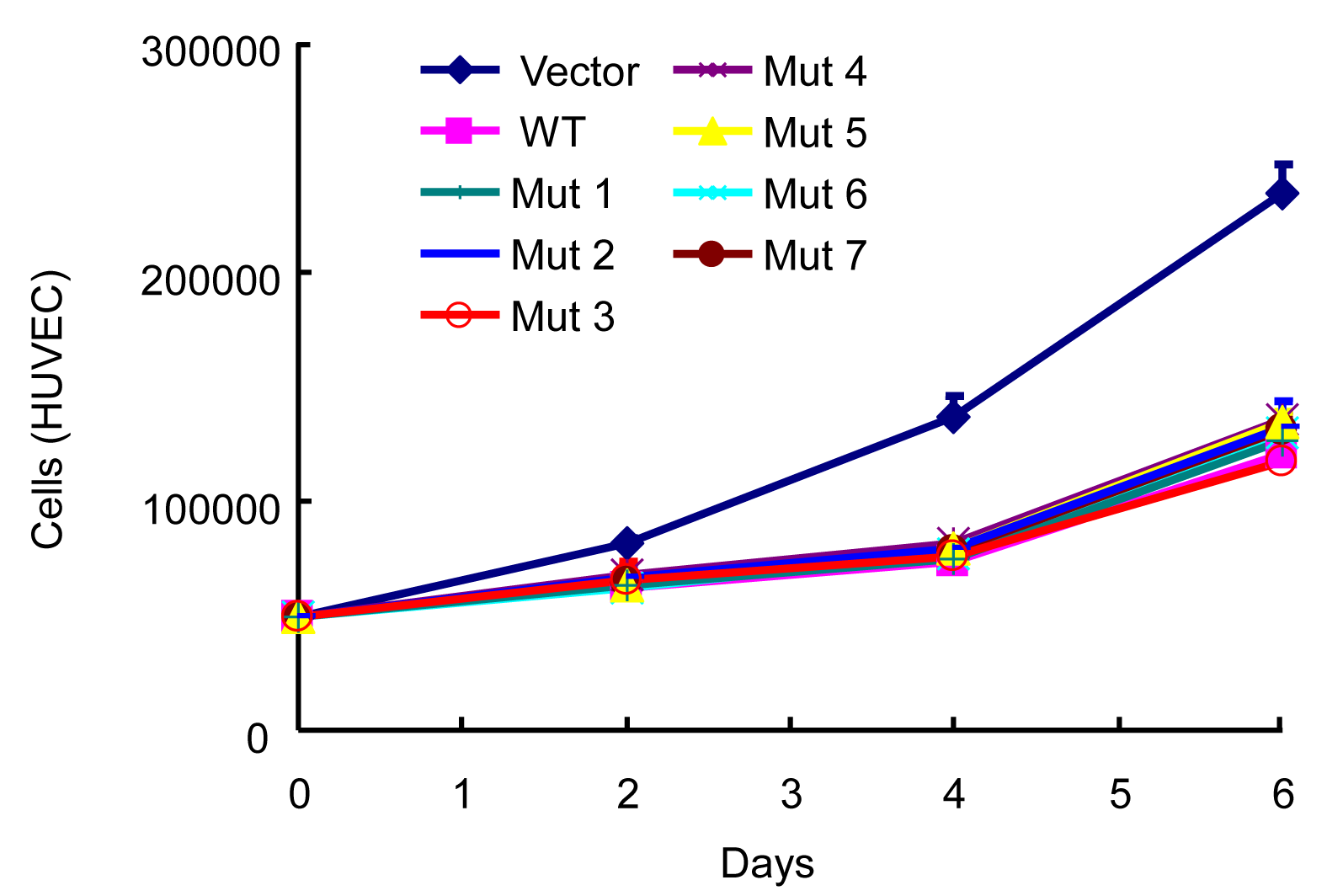


**Figure S1.** **Effect of wild-type DLC1 isoform 1 and mutants on HUVEC proliferation.** Wild-type DLC1 isoform 1 and mutants all showed a suppressive effect on cell proliferation compared to the control vector.


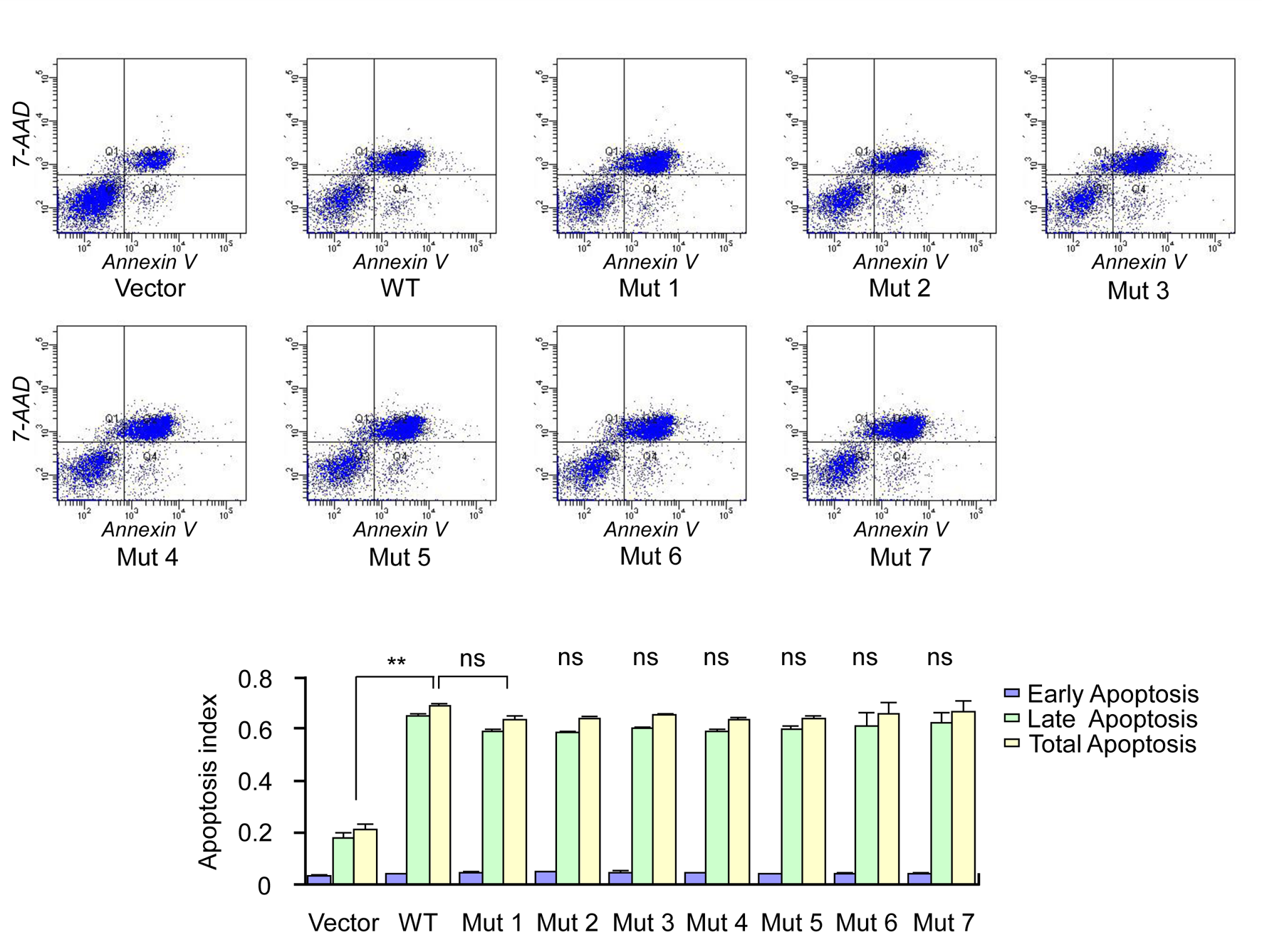


**Figure S2.** **The apoptosis analysis of wild-type DLC1 isoform 1 and mutants in HUVECs.** Top panel, representative images of the apoptosis analysis, as detected by flow cytometry; bottom panel, the quantification of cell apoptosis, as shown by the apoptosis index. Wild-type DLC1 isoform 1 and mutants all showed significant differences compared to the control vector. **Student’s t-test$P<0.01$. Ns, not significant.


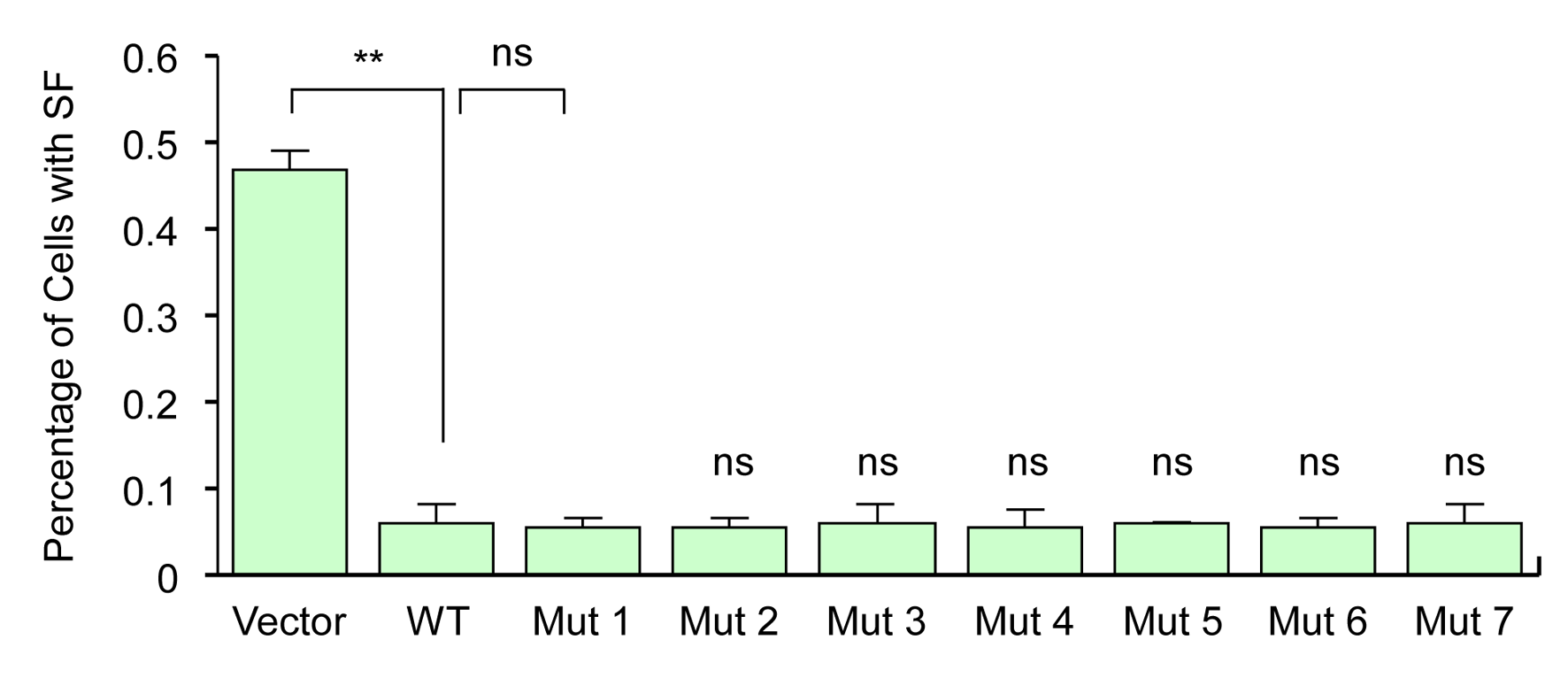


**Figure S3.** **Percentage of cells overexpressing wild-type DLC1 isoform 1 and mutants that exhibited stress fibers.** Wild-type DLC1 isoform 1 and mutants all showed significant differences compared to the control vector with regard to the suppression of stress fiber formation. **Student’s t-test$P<0.01$. Ns, not significant.


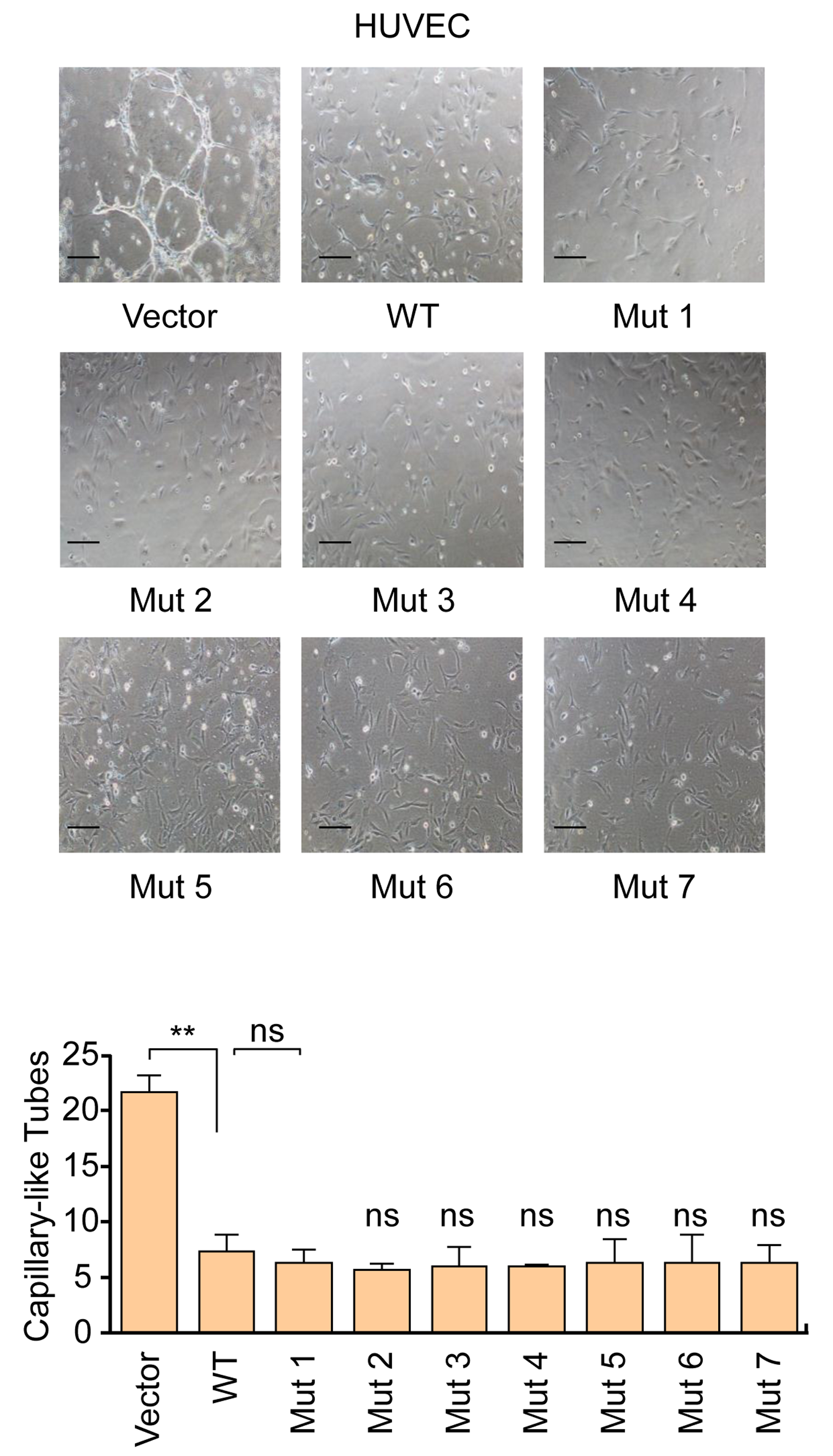


**Figure S4.** **Wild-type DLC1 isoform 1 and mutants had similar effects on angiogenesis.** Top panel, representative images of the tube-formation assay; bottom panel, quantification of angiogenesis, as shown by capillary-like tube counts. Wild-type DLC1 isoform 1 and mutants all exhibited the suppression of tube formation. **Student’s t-test$P<0.01$. Scale bars, 100 μm. Ns, not significant.
